# Prof. Remsen’s Report on Baltimore Water

**Published:** 1881-04

**Authors:** 


PROFESSOR REMSEN'S REPORT ON BALTIMORE WATER.—Prof. Ira Remsen, of the Johns Hopkins University, has sent his report to the Mayor of the city, from which we learn that neither the "dead fish," "crustacea," "algæ," nor "sulphuretted hydrogen" theories are sufficient to account for the execrable water supplied by our city works for several weeks past. He locates the cause, whatever it may be, in the reservoirs and pipes, as the lakes supplying them are filled with good water. Such being the case, no time should be lost in draining the reservoirs, and thoroughly cleansing the water pipes. A maniac saw fit to seek one of the most beautiful and picturesque of the former in which to shuffle off his mortal coil some time ago; and for aught we know to the contrary, other human remains may lie concealed from view beneath the placid surface of those beautiful waters! At all events, let us know as early as practicable, what there is that renders the water supplied by our hydrants so very offensive to the senses of taste and smell?

